# Melatonin Alleviates PM_2.5_-Induced Hepatic Steatosis and Metabolic-Associated Fatty Liver Disease in ApoE^−/−^ Mice

**DOI:** 10.1155/2022/8688643

**Published:** 2022-06-08

**Authors:** Zhou Du, Shuang Liang, Yang Li, Jingyi Zhang, Yang Yu, Qing Xu, Zhiwei Sun, Junchao Duan

**Affiliations:** ^1^Department of Toxicology and Sanitary Chemistry, School of Public Health, Capital Medical University, Beijing 100069, China; ^2^Beijing Key Laboratory of Environmental Toxicology, Capital Medical University, Beijing 100069, China; ^3^Core Facilities for Electrophysiology, Core Facilities Center, Capital Medical University, Beijing 100069, China

## Abstract

**Background:**

Exposure to fine particulate matter (PM_2.5_) is associated with the risk of developing metabolic-associated fatty liver disease (MAFLD). Melatonin is the main secreted product of the pineal gland and has been reported to prevent hepatic lipid metabolism disorders. However, it remains uncertain whether melatonin could protect against PM_2.5_-induced MAFLD.

**Methods and Results:**

The purpose of our study was to investigate the mitigating effects of melatonin on hepatic fatty degeneration accelerated by PM_2.5_ in vivo and in vitro. Histopathological analysis and ultrastructural images showed that PM_2.5_ induced hepatic steatosis and lipid vacuolation in ApoE^−/−^ mice, which could be effectively alleviated by melatonin administration. Increased ROS production and decreased expression of antioxidant enzymes were detected in the PM_2.5_-treated group, whereas melatonin showed recovery effects after PM_2.5_-induced oxidative damage in both the liver and L02 cells. Further investigation revealed that PM_2.5_ induced oxidative stress to activate PTP1B, which in turn had a positive feedback regulation effect on ROS release. When a PTP1B inhibitor or melatonin was administered, SP1/SREBP-1 signalling was effectively suppressed, while Nrf2/Keap1 signalling was activated in the PM_2.5_-treated groups.

**Conclusion:**

Our study is the first to show that melatonin alleviates the disturbance of PM_2.5_-triggered hepatic steatosis and liver damage by regulating the ROS-mediated PTP1B and Nrf2 signalling pathways in ApoE^−/−^ mice. These results suggest that melatonin administration might be a prospective therapy for the prevention and treatment of MAFLD associated with air pollution.

## 1. Introduction

Health risks associated with particulate air pollution have become a major focus of global concern due to rapid population growth, industrialization, and urbanization. Fine particulate matter at a size of ≤2.5 *μ*m (PM_2.5_) has been considered as a strong potential threat to public health that it can penetrate through the alveoli of lungs into the systemic circulation and accumulate in the liver, kidney, or brain [1–3]. Recently, a precise imaging technique was developed to visualize the deposition of PM_2.5_ particles in the liver through inhalation, providing solid evidence that the PM_2.5_ particles can enter the extrapulmonary organs [4]. Toxicological studies have demonstrated that the toxicity of PM_2.5_ not only induces respiratory and cardiovascular morbidities but also contributes to other unfavourable outcomes, such as systemic metabolic disorder, obesity, and the pathogenesis of metabolic-associated fatty liver disease (MAFLD) [2, 5], eventually resulting in liver dysfunction and damage. Consistent with evidence from animal studies, a prospective cohort study showed that people living in areas with higher PM_2.5_ concentrations had a 34% higher incidence of MAFLD than those living in areas with lower PM_2.5_ concentrations. The hazard ratio (HR) of MAFLD was 1.06 for every 1 *μ*g/m^3^ increase in PM_2.5_ [6]. MAFLD covers a broad spectrum of liver abnormalities from hepatic steatosis to inflammation and has become one of the main cause of cirrhosis and liver cancer. Its prevalence continues to progress universally, keeping pace with the obesity epidemic, reaching 20%-30% of the total population, 80–90% of obese individuals, and even more subjects with type 2 diabetes mellitus (T2DM) [7].

The mechanism for the pathophysiology of MAFLD was initially explained by the “two-hit” hypothesis. The first hit is that insulin resistance leads to enhanced hepatic de novo lipogenesis and decreased lipolysis. Mice exposed to PM_2.5_ have been demonstrated to develop MAFLD, characterized by changes in liver appearance, extensive distribution of lipid vacuoles, and balloon-like degeneration within the lobular structure [5, 8]. The accumulation of free fatty acid flux in hepatic cells further triggers a “second hit” involving oxidative stress and lipid peroxidation [9]. It has also been reported that PM_2.5_ exposure induces excessive oxygen species (ROS) production and redox homeostasis disorder [10, 11]. In brief, oxidative stress appears to be an integral mechanism that conveys hepatic injury in MAFLD and plays a well-described role in mediating the toxicity of PM_2.5_ [12]. However, the specific mechanism by which PM_2.5_ exposure promotes the risk of oxidative stress-driven MAFLD remains incompletely understood.

A growing body of evidence in the cellular and molecular biology of lipid metabolism have shown that protein tyrosine phosphatase 1B (PTP1B) is a new activator in the process of MAFLD that regulates lipogenesis in the liver [13, 14]. Total PTP1B protein levels were generally upregulated in liver biopsies from patients with MAFLD [15]. Functionally, PTP1B deficiency prevents the adverse metabolic effects of a high-fat diet, including weight gain, increased liver lipids, and reduced glucose tolerance [16]. PTP1B^−/−^ mice also exhibited downregulation of genes involved in fat production, including sterol regulatory element-binding proteins (SREBPs) [17]. Furthermore, SREBP-1 could extensively affect multiple metabolic steps in the liver and extranet, thereby regulating the progression of MAFLD [18]. However, it is still not clear whether PM_2.5_ has a targeted regulatory effect on PTP1B.

Melatonin is well-known for its ability to neutralize ROS and reduce oxidative stress [19]. It upregulates nuclear factor erythroid 2-related factor 2 (Nrf2) through inhibition of Kelch—like ECH-associated protein (Keap1) to suppress oxidative stress in the liver [20]. Investigations have noted that melatonin critically participates in lipid metabolism and potentially contributes to the onset and progression of MAFLD [21, 22]. However, it remains uncertain whether melatonin could protect against PM_2.5_-induced oxidative stress in the liver and ameliorate MAFLD.

Compared with the general population, people with obesity, hyperlipidaemia, or abnormal lipid metabolism are more sensitive to PM_2.5_ and have a higher risk of developing MAFLD [23, 24]. According to a cohort study of full-exome association of alanine aminotransferase, ApoE was found closely linked with fatty liver [25], and allele-specific variants of ApoE were associated with an increased incidence of MAFLD and obesity [26]. Thus, intense efforts have been made to investigate MAFLD based on ApoE^−/−^ mice [27–30]. Hua et al. demonstrated that naringin administration improved metabolic parameters in ApoE^−/−^ mice, inhibited hepatic steatosis, and reduced hepatic fibrosis [31]. Stachowicz et al. found that high fat diet resulted in more exacerbated hepatic steatosis in ApoE^−/−^ mice [32]. In this study, ApoE^−/−^ mice were chosen as an animal model to explore the molecular mechanism of the melatonin-mediated protective effects against PM_2.5_-induced MAFLD. We speculated that melatonin may ameliorate PM_2.5_-induced MAFLD. Our findings supported this hypothesis and further indicated that melatonin alleviated the disturbance of PM_2.5_-triggered hepatic steatosis and liver damage by regulating the ROS-mediated PTP1B and Nrf2 signalling pathways. These results not only provide novel insight into the underlying molecular mechanism by which PM_2.5_ contributes to the pathogenesis of MAFLD but also suggest the use of melatonin as a potential treatment.

## 2. Materials and Methods

### 2.1. Collection and Extraction of PM_2.5_

PM_2.5_ was collected on quartz fibre filters with a special sampler (TH-1000C, Wuhan Tianhong, China) from Capital Medical University (Beijing, China) for the entire year of 2017. The physicochemical characterization of PM_2.5_ was described in detail in our previous study. Tables S1 and S2 show the results of element analysis. S, Ca, Na, Si, and Fe are the most abundant elements. Toxic heavy metals (including Mn, Cd, Cr, Ni, and Sb), toxic nonmetallic elements (As), and water-soluble ions (NO_3_^−^, SO4_2_^−^, and NH4^+^) were detected in PM_2.5_ [33, 34]. Sampled filters were placed in ultrapure water for 3 hours using an ice-water bath ultrasonic instrument. Then, freeze-dried samples were irradiated with ultraviolet light for 2 hours, diluted and mixed with pure water, and suspended in PM_2.5_ by ultrasonication for 30 minutes for later use.

### 2.2. Animals and Treatments

Seven-week-old male ApoE^−/−^ mice (specific-pathogen free) were obtained from the Experimental Laboratory Animal Technology Co., Ltd. (Vital River, Beijing, China). Animal experimental procedures were approved by the Experimental Animal Welfare Committee (Capital Medical University; AEEI-2016-076). All mice were fed a high-fat diet (0.15% cholesterol and 21% fat). After acclimatization for one week, a total of 60 mice were randomly divided into four groups: (i) control group (Con): animals were treated with saline; (ii) PM_2.5_ group (PM_2.5_): animals were treated with PM_2.5_; (iii) melatonin group (Mel): animals were treated with melatonin; and (iv) melatonin and PM_2.5_ group (PM_2.5_+Mel): animals were treated with PM_2.5_ and melatonin. Mice were orally gavaged with melatonin (20 mg/kg•bw, in 20~25 *μ*L of 0.5% ethanol solution) daily and PM_2.5_ (5 mg/kg•bw, in 20~25 *μ*L of saline) via intratracheal instillation twice a week for 4 weeks. The control mice received a corresponding volume of blank filters eluted with saline by intratracheal instillation. The vehicle mice were gavaged with the same amount of sterile water (0.5% ethanol).

According to the concentration and intervention method of melatonin in previous studies, melatonin (Sigma, USA) was dissolved in absolute ethanol and diluted in sterile water to a final concentration of 0.5% ethanol, with the oral gavage at a dose of 20 mg/kg/day [35, 36]. The dose of PM_2.5_ exposure was based on the respiratory physiological parameters of mice and the annual mean PM_2.5_ concentration (35 *μ*g/m^3^), according to the WHO air quality guidelines [37]. The respiratory volume of an adult mouse (25 g) is 0.15 mL at each breath, and the breath rate is 163 times per min, and respiratory volume for one day reaches 0.035208 m^3^. For this reason, the daily exposure of mice was 0.035208∗35 *μ*g/m^3^ = 1.23228 *μ*g. Based on the body weight of mice 25 g and the extrapolation coefficient of species 100, the volume of intratracheal instillation was 1.23228 *μ*g/25 g∗100 = 4.93 *μ*g/g. Previous studies have demonstrated that PM_2.5_ at 5 mg/kg can cause varying degrees of organ damage [38, 39]. Therefore, a dose of 5 mg/kg was selected for animal modeling.

### 2.3. Ultrasonic Examination of Liver

Before ultrasound imaging, the mice were fasted for 12 h, the abdominal regions were shaved, and then the mice were anaesthetized with a saturated tribromoethanol solution via intraperitoneal injection. We acquired transcutaneous ultrasound images using a Vevo2100 Ultrasonic Doppler System (Fujifilm Visual Sonics, US).

### 2.4. Histopathological Examination

Both haematoxylin-eosin (H&E) and Oil Red O staining are effective and reproducible methods for quantifying hepatic steatosis [40]. For histological examination, liver specimens were fixed overnight with 4% paraformaldehyde and then embedded in paraffin sections (4–6 *μ*m). Tissue sections were counterstained with H&E. To visualize lipid droplet accumulation, frozen liver sections (10 *μ*m) were taken, stained with Oil Red O (0.5%) for 10 min, washed and rinsed with isopropanol, and counterstained with haematoxylin for a few seconds. Representative photographs were taken at 200x and 400x magnification using an in-microscope system. There were 6 samples in each group, and twenty regions were randomly selected from each separate section. The “color picker” in Image-Pro-Plus was used to select the red fat droplets in images until all the red fat droplets were marked. Then, Oil Red O-stained area was measured, and its ratio to the total tissue area was calculated.

### 2.5. Ultrastructural Observation by Transmission Electron Microscopy (TEM)

Lipid accumulation in the liver tissue was observed by transmission electron microscopy. The liver tissues were immediately placed into 2.5% glutaraldehyde for 10 min at 4°C and then washed with PBS 3 times and dehydrated. Sample sections (60 nm) were stained on copper mesh and assessed using TEM (JEM-2100plus).

### 2.6. Detection of ROS Levels in Liver Tissue

Frozen sections of the liver were washed, DHE solution (10 *μ*M) was added, and the sections were incubated at room temperature for 30 min. A confocal microscope (LSCM, TCS SP8 STED, Germany) was used to capture fluorescence images. There were 6 samples in each group, and 3 visual fields were randomly selected for each sample. Then, the ratio of red area to total area was statistically analyzed by Image-Pro-Plus.

### 2.7. Cell Culture and Treatment

The human normal liver cell line L02 was obtained from Shanghai Institutes for Biological Sciences (SIBS, China). Cells were cultured in Dulbecco's modified Eagle's medium (DMEM; Corning, USA) containing 1% penicillin-streptomycin solution and 10% foetal bovine serum (Corning, USA) at 37°C in a humidified incubator with 5% CO_2_. Palmitic acid (PA) is an inducer for cell steatosis. For treatment before each experiment, cells were treated with PA solution dissolved in DMEM for 24 h. When the cell density reached 70%-80%, DMEM (without serum) containing PM_2.5_ or melatonin was added and then cultured for 24 h. The control group was cultured in a constant volume of pure medium.

### 2.8. Assessment of Cytotoxicity

A total of 1 × 10^4^ L02 cells per well were seeded in 96-well culture plates. When the cell density reached 50%, the L02 cells were exposed to gradient concentrations of PM_2.5_ (0, 12.5, 25, and 50 100 *μ*g/mL), PA (0, 50, 100, 200, 400, 800, and 1600 *μ*mol/L), and melatonin (0, 12.5, 25, 50, 100, and 200 *μ*mol/L). According to the protocols, cell viabilities were measured by Cell Counting Kit-8 (CCK-8, Tongren, Japan), and the absorbance was measured at 450 nm using a microplate reader (Thermo, USA).

### 2.9. Biochemical Parameter Analysis

Triacylglycerols (TAGs), total cholesterol (TC), low-density cholesterol (LDL-C), high-density cholesterol (HDL-C), glutathione peroxidase (GSH-Px), superoxide dismutase (SOD), and malonaldehyde (MDA) levels were measured spectrophotometrically according to the instructions of the kit (Nanjing Jiancheng Institute of Biotechnology, Nanjing, China). Protein concentration was determined using a BCA protein assay kit (Dingguo Changsheng Biotech, China). The 4-hydroxynonenal (4-HNE) activity was determined using a Hailian Biotechnology Co. Ltd. ELISA (enzyme-linked immunosorbent assay) kit (Jiangxi, China).

### 2.10. Cellular BODIPY Staining

BODIPY™ 493/503 (Thermo, USA) is a lipophilic fluorescent probe targeting polar lipids that can be used to label cell neutral lipid content, especially lipid content localized to lipid droplets. It was dissolved in anhydrous ethanol to generate a 10 mM stock solution, which was frozen, dried, and stored away from light. PBS was used to dilute the solution to 10 *μ*M, which was used for incubation with the cells at room temperature in the dark for 20 min; finally, the cells were observed via a confocal microscope (LSCM, TCS SP8 STED, Germany).

### 2.11. Detection of ROS Levels in L02 Cells

The level of ROS in L02 cells was analyzed by flow cytometry. After the cells were infected for 24 h, a 2′,7′-dichlorofluorescein diacetate (DCFH-DA, Sigma, USA) working solution (10 *μ*M) was added followed by incubation at 37°C for 30 min. The cells were washed twice with PBS, and ROS levels were determined by flow cytometry. The single-parameter histograms were obtained by taking the logarithm of fluorescence signal as abscissa and the number of cells as ordinate, which could intuitively reflect the relative intensity of ROS in living cells. The average fluorescence intensity was the number of cells divided by the area under each peak. ROS fluorescence was measured with a confocal scanning laser microscope. To quantify the ROS production in L02 cells treated with PM_2.5_ and/or melatonin, cells were pretreated with the ROS inhibitor N-acetylcysteine (NAC; Sigma, USA) (1 mM) for 1 h before PM_2.5_ and/or melatonin exposure.

DCFH-DA, intracellular reactive oxygen species detection probe, is a universal indicator of oxidative stress. After it enters the cell, it is hydrolyzed to produce DCFH. Intracellular reactive oxygen species can oxidize nonfluorescent DCFH to produce fluorescent DCF. Intracellular reactive oxygen species (ROS) levels were obtained by measuring the fluorescence intensity of DCF.

### 2.12. Real-Time Polymerase Chain Reaction (qPCR)

Total RNA from L02 cells and liver tissue was extracted using TRIzol™ Reagent (Thermo, USA). According to the protocol, the RNA was reverse transcribed into cDNA with PrimeScript RT Master Mix (Takara, China). SYBR® Premix Ex Taq™ II (Takara, China) was used for quantitative PCR on a Realplex2 (Eppendorf, Germany). The mRNA primers are listed in Table 1.

### 2.13. Western Blot Analysis

Protein extracts of mouse liver tissue and L02 cells were prepared by a Whole Cell Lysis Assay Kit (Keygen Biotech, China), and the concentrations were determined by a BCA protein quantitative assay kit (Dingguo Changsheng Biotech, China). The same amounts of protein were separated by electrophoresis using 8%–12% SDS-PAGE gels and transferred to suitably sized nitrocellulose membranes (Pall Corp., USA). After blocking with 5% BSA or 5% skim milk in Tris-buffered saline (TBS), the membranes were incubated with primary antibodies at 4°C overnight, including PTP1B (Abcam, UK), SP1 (Abcam, UK), P-SP1 (Abcam, UK), SOD (Abcam, UK), Nrf2 (Abcam, UK), Keap1 (Abcam, UK), PP2A (Santa, USA), P-PP2A (Santa, USA), SREBP-1 (Santa, USA), and GAPDH (CST, USA). The next day, the membranes were washed three times with TBST and incubated with an anti-rabbit/mouse IgG secondary antibody (CST, USA). A LI-COR Odyssey system (LI-COR Biosciences, USA) was used for detection of the protein bands, which were quantified using Image Studio software (NIH, Bethesda, MD).

### 2.14. The Addition of PTP1B Inhibitor

We dissolve PTP1B inhibitor PTP1B-IN-1 (MedChemExpress, USA) in DMSO to prepare a stock solution at a concentration of 20 mM. Then, L02 cells were treated with PTP1B-IN-1 (5 *μ*g/mL, 10 *μ*g/mL, 20 *μ*g/mL, and 40 *μ*g/mL) diluted with DMEM for 12 h. After qPCR analysis, 10 *μ*g/mL was selected as the dose of PTP1B-IN-1.

### 2.15. Statistical Analysis

SPSS 24.0 software was used to analyze all experimental data. Data are presented as the mean ± SD. Data consistent with a normal distribution and an equal variance were tested by one-way ANOVA or two-way ANOVA. Among them, the data with only one variable of PM_2.5_ adopted one-way ANOVA, and the data with two variables of melatonin and PM_2.5_ adopted two-way ANOVA. The Kruskal-Wallis test was used for nonparametric data. A value of *p* < 0.05 indicates statistical significance. Each experiment was repeated at least three times.

## 3. Results

### 3.1. Melatonin Alleviated the PM_2.5_-Induced Fatty Increase and Steatosis in ApoE^−/−^ Mice

**Figure 1:**
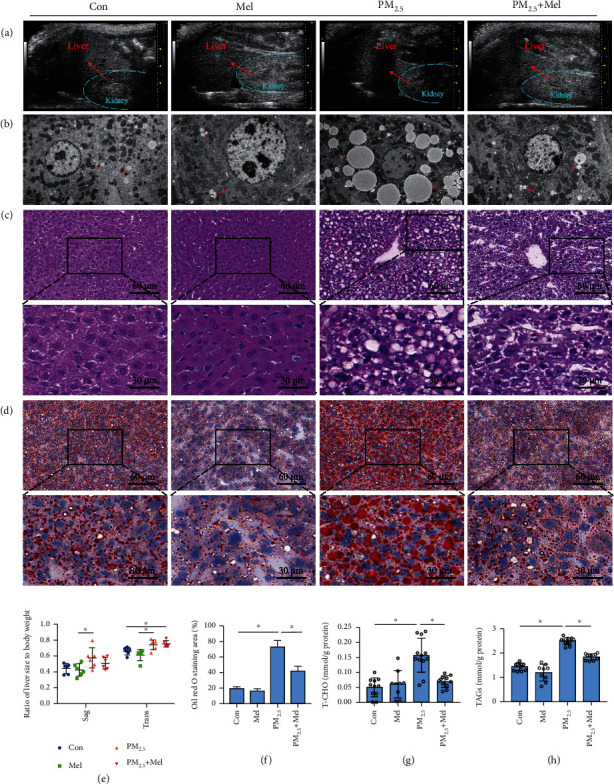
Melatonin improved the increased lipid content and steatosis in the liver induced by PM_2.5_. (a) Ultrasound examination of liver—comparison of liver echo and kidney echo. (b) The ultrastructure of liver tissues via electron microscopy (magnification, 200; scale bar, 2 *μ*m). (c) Liver sections with haematoxylin and eosin (H&E) staining (magnification, 200 and 400; scale bar, 60 *μ*m and 30 *μ*m). (d) Liver steatosis assessed by Oil Red O staining (magnification, 200 and 400; scale bar, 60 *μ*m and 30 *μ*m). (e) Liver sag (anterior-posterior diameter) and liver trans (left-right diameter) measurement to mice weight ratio. (f) The ratio of the Oil Red O-stained area to the total tissue area. (g) Hepatic total cholesterol lipid levels (mmol/g). (h) Hepatic triacylglycerol lipid levels (mmol/g). Con: animals were treated with saline; Mel: animals were treated with melatonin; PM_2.5_: animals were treated with PM_2.5_; PM_2.5_+Mel: animals were treated with melatonin and PM_2.5_. Data are shown as means ± SD. *n* = 6 − 12 mice per group. ^∗^*P* < 0.05 for Con group vs PM_2.5_ group and PM_2.5_ group vs PM_2.5_+Mel group.

To evaluate the effects of PM_2.5_ on liver lipid accumulation in mice, we first confirmed that PM_2.5_ induced liver changes by ultrasound examination. The contrast of liver-kidney echo is one of the obvious manifestations of a fatty liver. Compared to the control group, ultrasonography showed that the echo of the liver parenchyma was high and dense, and the contrast sign of the liver and kidney was positive in the PM_2.5_ group, but this expression was relieved in the melatonin group (Figure 1(a)). The anterior-posterior diameter and left-right diameter of the liver can reflect changes in liver size. Although these two indicators did not change significantly between the PM_2.5_ and control groups, there was a significant difference between the PM_2.5_ and melatonin groups (Figure 1(e)). Concordant with this, analysis of the body weights, liver weights, and liver coefficient of the mice showed that the liver size of the PM2.5 group was significantly higher than that of the control group, and that melatonin had a slight alleviating effect on liver weight gain (Supplementary Figure S1E-1G). Histological examinations of the liver are presented in Figures 1(b)–1(d). Electron microscopy images showed large lipid droplets, and HE and Oil Red O staining revealed notably enlarged adipocytes, fatty degeneration, and specific lipid accumulation in PM_2.5_-treated mice compared to the control group. However, treatment with melatonin visibly alleviated these alterations. Next, changes in lipid content in the liver were examined. The levels of total cholesterol (TC) and triacylglycerols (TAGs) in the livers increased in response to PM_2.5_, while melatonin treatment significantly decreased lipid levels (Figures 1 (g)and 1(h)). In addition, the Masson staining and qPCR analysis results of inflammatory factors (IL-1, IL-6, and TNF-*α*) in liver tissue showed that PM_2.5_ could cause liver injury, and melatonin had a mitigating effect (Supplementary Figure S1A-1D). Taken together, these results suggested that PM_2.5_ exposure could induce hepatic lipid metabolism disorders and that melatonin treatment had a beneficial effect on the liver.

### 3.2. Protective Effects of Melatonin on PM_2.5_-Induced Oxidative Damage in Liver

**Figure 2:**
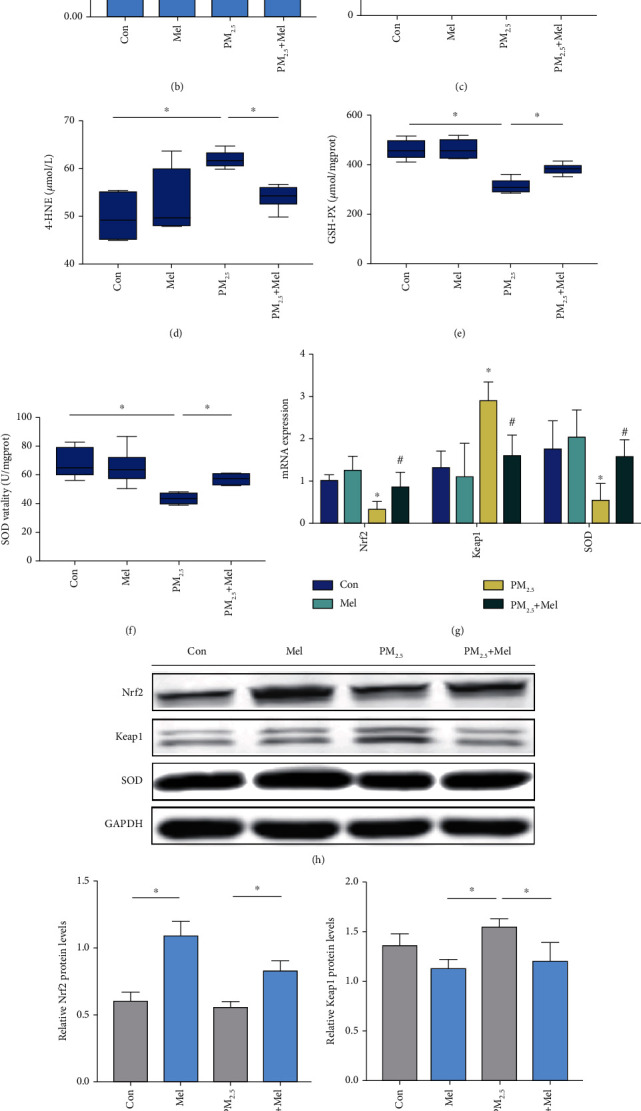
Melatonin improved liver oxidative damage induced by PM_2.5_. (a) Production of ROS detected by the fluorescent probe DHE (magnification, 200; scale bar, 20 *μ*m). (b) Quantitative analysis of ROS production is reflected by the mean fluorescence intensity as shown in different groups. (c) The level of MDA. (d) The level of 4-HNE. (e) The level of GSH-PX. (f) The vitality of SOD. (g) The mRNA expression of Nrf2, Keap1, and SOD. (h) Western blotting of Nrf2, Keap-1, and SOD. (i) Protein quantification of Nrf2. (j) Protein quantification of Keap1. (k) Protein quantification of SOD. All values are presented as the mean ± SD (*n* = 6). ^∗^*P* < 0.05 for Con group vs PM_2.5_ group and ^#^*P* < 0.05 for PM_2.5_ group vs PM_2.5_+Mel group.

Multiple studies have shown that PM_2.5_ aggravates lipid metabolism disorder by inducing oxidative stress. To determine the effects of PM_2.5_/melatonin on ROS production, liver sections were stained with the fluorescent probe DHE to evaluate ROS levels. As shown in the representative fluorescence micrographs (red fluorescence) and histogram of ROS relative fluorescence density (Figures 2(a) and 2(b)), PM_2.5_ treatment increased ROS generation, while melatonin supplementation alleviated ROS generation. MDA and 4-HNE are important indexes of lipid peroxidation. According to the quantitative analysis, exposure to PM_2.5_ resulted in significantly increased levels of MDA and 4-HNE, whereas melatonin treatment reversed these effects (Figures 2(c) and 2(d)). Moreover, the degree of oxidative stress was detected by examination of GSH-Px and SOD. As anticipated, PM_2.5_ treatment reduced the activity of GSH-Px and SOD in the liver compared to the control. However, these two indicators were reversed by melatonin (Figures 2(e) and 2(f)). Subsequent analysis of the protein and mRNA expression levels of known indicators of oxidative stress, including Keap1, Nrf2, and SOD, was performed. In the present study, PM_2.5_ exposure decreased Nrf2 and SOD mRNA expression and increased Keap1 mRNA expression, and these negative effects were mitigated by melatonin (Figure 2(g)). Consistently, compared with the control group, the expression of Nrf2/Keap1 and the SOD protein in the PM_2.5_ group was not significantly different, but there were significant changes after melatonin treatment (Figures 2(h)–2(k)). However, this result did not rule out an impact on their gene expression. Overall, these data suggested that the antioxidative stress effects of melatonin might protect the liver from PM_2.5_ exposure.

### 3.3. Melatonin Ameliorated Abnormal Liver Lipid Metabolism and Caused Elevated PTP1B Expression Induced by PM_2.5_

**Figure 3:**
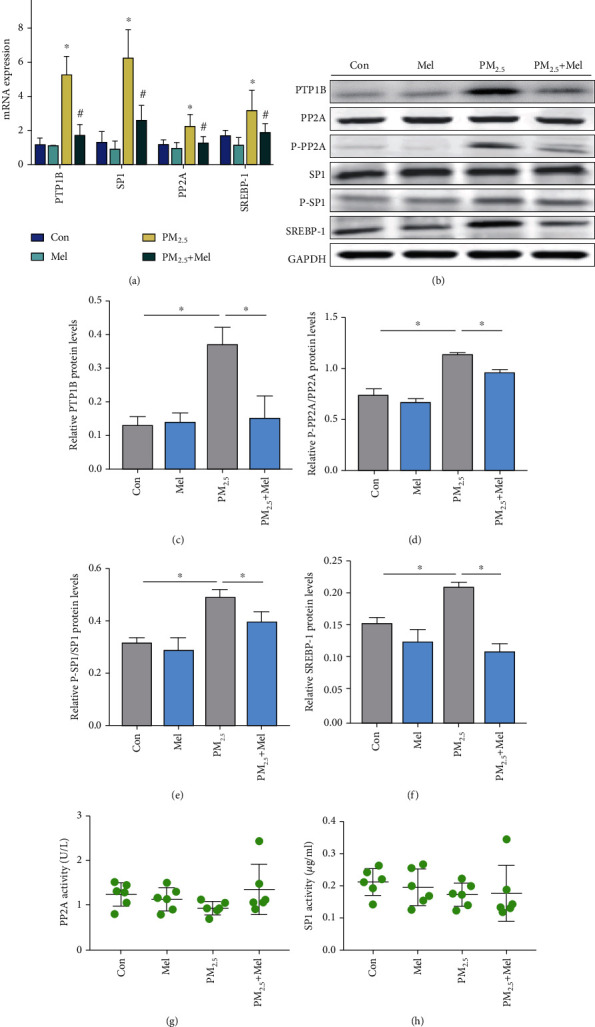
Melatonin ameliorated abnormal liver lipid metabolism caused by elevated PTP1B expression induced by PM_2.5_. (a) The mRNA expression of PTP1B, PP2A, SP1, and SREBP-1. (b) Western blotting of PTP1B, PP2A, P-PP2A, SP1, P-SP1, and SREBP-1. (c) Protein quantification of PTP1B. (d) Protein quantification of P-PP2A/PP2A. (e) Protein quantification of P-SP1/SP1. (f) Protein quantification of SREBP-1. (g) The activity of PP2A. (h) The activity of SP1. All values are presented as the mean ± SD (*n* = 6). ^∗^*P* < 0.05 for Con group vs PM_2.5_ group and ^#^*P* < 0.05 for PM_2.5_ group vs PM_2.5_+Mel group.

To identify the potential mechanisms by which PM_2.5_ or melatonin induced gene expression in lipid accumulation, qPCR analysis of genes related to lipid metabolism was examined in liver samples. Analysis of the mRNA expression levels showed that PM_2.5_ exposure was associated with lipid metabolism disorder, and PTP1B, which is closely related to MAFLD, was an important upregulated transcription factor (Supplementary Figure S2). PTP1B is a key regulator of the antioxidant system and an activator of liver adipogenesis. Next, the regulation of downstream PTP1B on genes related to liver lipid metabolism was verified by qPCR analysis. As expected, PM_2.5_ exposure significantly increased the expression of lipid accumulation markers (PP2A, SP1, and SREBP-1), whereas exogenous melatonin treatment decreased their levels (Figure 3(a)). Lipid accumulation plays an important role in the progression of MAFLD. Additionally, for further verification, Western blotting was carried out. PM_2.5_ administration led to an increase in the accumulation of PTP1B, P-PP2A/PP2A, P-SP1/SP1, and SREBP-1, and subsequent analysis showed that melatonin inhibited their expression (Figures 3(b)–3(f)). However, the enzymatic activities of PP2A and SP1 did not change significantly in PM_2.5_-exposed mice (Figures 3(g) and 3(h)). These results could be greatly downregulated by melatonin treatment. All of the above data indicated that melatonin supplementation reduced adiposity accumulation triggered by PM_2.5_.

### 3.4. PM_2.5_ Exposure Caused Lipid Accumulation in L02 Cells by Inducing ROS Production

**Figure 4:**
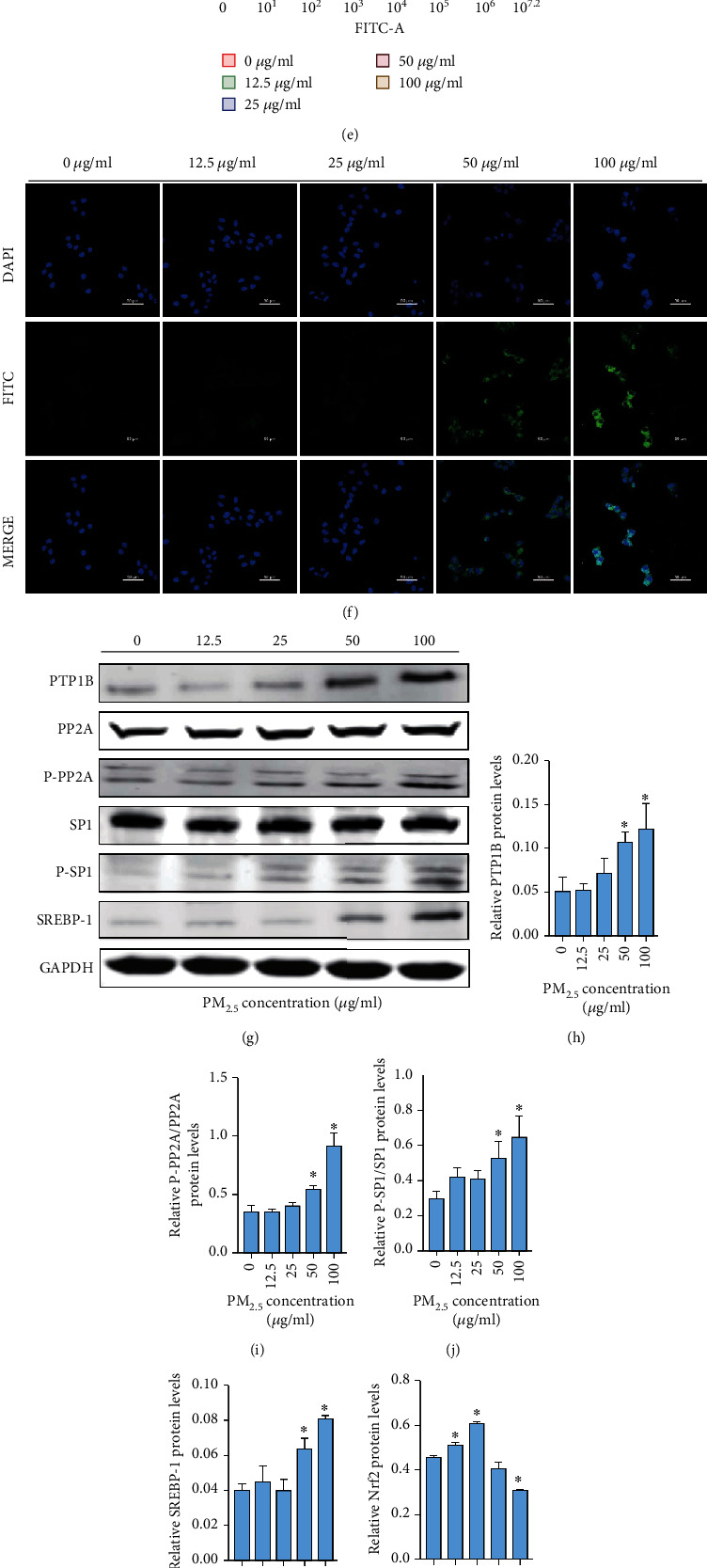
PM_2.5_ induced lipid accumulation in hepatocytes. (a) Cell viability. (b) Total cholesterol lipid levels (mmol/g). (c) Triacylglycerol lipid levels (mmol/g). (d) Representative fluorescence intensity images obtained from flow cytometry in L02 cells. (e) Analysis of fluorescence intensity obtained from flow cytometry. (f) Representative confocal images of ROS. (g) Western blotting of PTP1B, PP2A, P-PP2A, SP1, P-SP1, and SREBP-1. (h) Protein quantification of PTP1B. (i) Protein quantification of P-PP2A/PP2A. (j) Protein quantification of P-SP1/SP1. (k) Protein quantification of SREBP-1. (l) Protein quantification of Nrf2. (m) Protein quantification of Keap1. (n) Protein quantification of SOD. (o) Western blotting of Nrf2, Keap1, and SOD. All values are presented as the mean ± SD. ^∗^*P* < 0.05.

The cytotoxic effects of PM_2.5_ on L02 cell viability was assessed by CCK-8 assay. As evidenced in Figure 4(a), the viability of L02 cells decreased with increasing doses of PM_2.5_. Treatment with between 25 and 100 *μ*g/mL PM_2.5_ for 24 h showed significant differences compared with untreated cells. To detect the effects of PM_2.5_ on lipid synthesis, L02 cells were exposed to various doses of PM_2.5_ (0–100 *μ*g/mL) for 24 h. As expected, the contents of T-CHO and TAGs in L02 cells gradually increased as the concentration of PM_2.5_ increased (Figures 4(b) and 4(c)). Next, ROS generated by PM_2.5_ treatment were detected by flow cytometry analysis quantification and confocal microscopy (Figures 4(d)–4(f)). Compared to the control group, the ROS fluorescence intensity was observably increased in PM_2.5_-treated L02 cells, which occurred in a manner dependent on the PM_2.5_ concentration. Then, activation of the PTP1B pathway was determined after PM_2.5_ exposure. Quantitative measurements of protein expression showed that P-PP2A/PP2A, P-SP1/SP1, and SREBP-1 expression was dependent on the PM_2.5_ concentration (Figures 4(g)–4(k)). In addition, activation of the Nrf2/Keap1 pathway was detected by Western blot. Low-dose PM_2.5_ induced the upregulation of Nrf2 and SOD protein expression, while high-dose PM_2.5_ inhibited their expression. The opposite trend was observed for Keap1 (Figures 4(l)–4(o)). Given the above data, although the effect indexes in the 25 *μ*g/mL dose group were significantly different, the expression of the proteins in the PTP1B pathway was significantly different in the 50 *μ*g/mL dose group, and the Nrf2/Keap1 pathway was inhibited in the 50 *μ*g/mL dose group. For this reason, the concentration of PM_2.5_ (50 *μ*g/mL) was selected for subsequent experiments.

**Figure 5:**
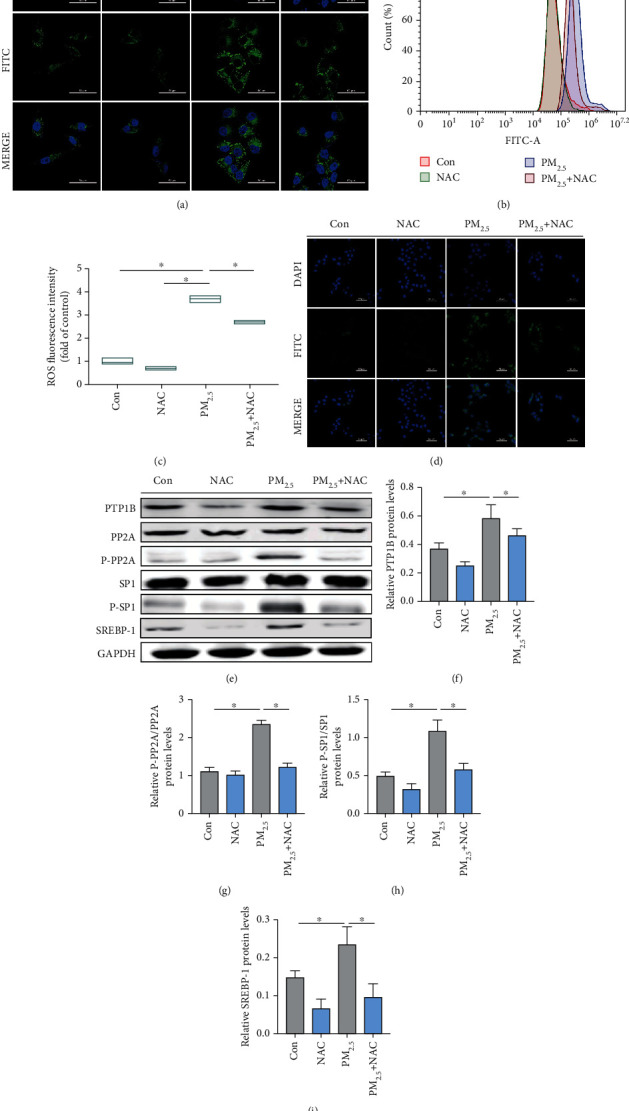
PM_2.5_ induced lipid accumulation in hepatocytes by increasing ROS levels and PTP1B expression. (a) BODIPY staining of L02 cells (scale bar, 50 *μ*m). (b) Representative fluorescence intensity images obtained from flow cytometry in L02 cells. (c) Analysis of fluorescence intensity obtained from flow cytometry. (d) Representative confocal images of ROS. (e) Western blotting of PTP1B, PP2A, P-PP2A, SP1, P-SP1, and SREBP-1. (f) Protein quantification of PTP1B. (g) Protein quantification of P-PP2A/PP2A. (h) Protein quantification of P-SP1/SP1. (i) Protein quantification of SREBP-1. All values are presented as the mean ± SD. ^∗^*P* < 0.05.

To obtain more details on the role of oxidative stress in PM_2.5_-induced liver lipid accumulation, NAC (N-acetylcysteine) was added to L02 cells exposed to PM_2.5_ (Figure 5). Representative images of BODIPY staining are shown in Figure 5(a). NAC treatment significantly abolished the PM_2.5_-induced increase in lipid content in L02 cells. Flow cytometry and confocal microscopy data analysis indicated that the fluorescence intensity of the ROS generated in the NAC-treated cultures exposed to PM_2.5_ (50 *μ*g/mL) was significantly less than that in PM_2.5_-exposed cells (Figures 5(b)–5(d)). Additionally, NAC treatment restored the expression of the proteins PTP1B, P-PP2A/PP2A, P-SP1/SP1, and SREBP-1 in PM_2.5_-treated L02 cells to levels comparable with the control (Figures 5(e)–5(i)). PM_2.5_ exposure initially triggered oxidative stress, which further led to lipid peroxidation. In short, the effects of PM_2.5_ exposure on lipid accumulation in L02 cells were ROS dependent and involved PTP1B signalling.

### 3.5. Melatonin Alleviated PM_2.5_-Induced Oxidative Damage and Lipid Accumulation In Vitro

**Figure 6:**
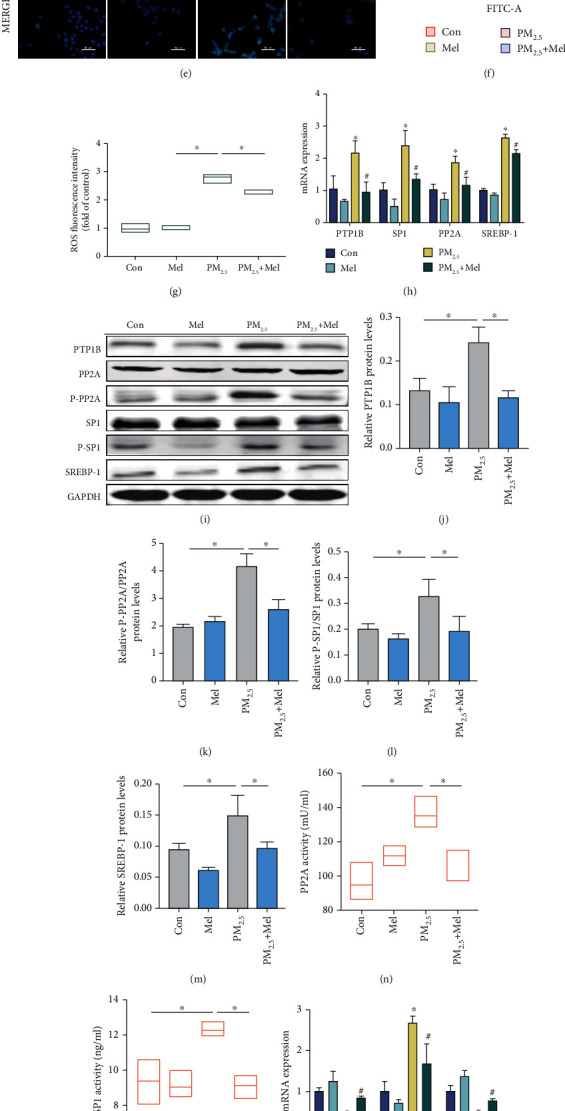
Melatonin alleviated PM_2.5_-induced oxidative damage and upregulated PTP1B expression in vitro. (a) and (b) Cell ability. (c) Total cholesterol lipid levels (mmol/g). (d) Triacylglycerol lipid levels (mmol/g). (e) Representative confocal images of ROS. (f) Representative fluorescence intensity images obtained from flow cytometry in L02 cells. (g) Analysis of fluorescence intensity obtained from flow cytometry. (h) The mRNA expression of PTP1B, PP2A, SP1, and SREBP-1. (i) Western blotting of PTP1B, PP2A, P-PP2A, SP1, P-SP1, and SREBP-1. (j) Protein quantification of PTP1B. (k) Protein quantification of P-PP2A/PP2A. (l) Protein quantification of P-SP1/SP1. (m) Protein quantification of SREBP-1. (n) The activity of PP2A. (o) The activity of SP1. (p) The mRNA expression of Nrf2, Keap-1, and SOD. (q) Western blotting of Nrf2, Keap-1, and SOD. (r) Protein quantification of Nrf2. (s) Protein quantification of Keap-1. (t) Protein quantification of SOD. All values are presented as the mean ± SD. ^∗^*P* < 0.05 for Con group vs PM_2.5_ group and ^#^*P* < 0.05 for PM_2.5_ group vs PM_2.5_+Mel group.

To determine the dosage of melatonin to be used, CCK-8 assays were applied to examine cell viability. With increasing concentrations of melatonin, the viability of L02 cells first increased and then decreased, and 200 *μ*mol/L melatonin showed a significant decrease compared to the control group (Figure 6(a)). Moreover, the effects of PM_2.5_ and melatonin on cell viability were investigated (Figure 6(b)). Finally, 100 *μ*mol/L melatonin was selected for further experiments. First, the protective effects of melatonin on lipid accumulation were detected by measuring the levels of TC and TAGs (Figures 6(c) and 6(d)). The results indicated that melatonin decreased the lipid levels in PM_2.5_-induced L02 cells compared with PM_2.5_ treatment alone. Flow cytometry and confocal microscopy data analysis indicated that treatment with melatonin could restore ROS to control levels (Figures 6(e)–6(g)). Furthermore, melatonin altered the mRNA levels of PTP1B, PP2A, SP1, and SREBP-1 in the presence of PM_2.5_ (Figure 6(h)). Similarly, the levels of the proteins in the PTP1B pathway showed that melatonin reduced PM_2.5_-induced lipid accumulation (Figures 6(i)–6(m)). Intriguingly, melatonin restored the PM_2.5_-mediated increase in PP2A and SP1 activity to a normal level (Figures 6(n) and 6(o)). Moreover, PM_2.5_ exposure decreased the Nrf2 and SOD mRNA expression and increased the Keap1 mRNA expression, and these negative effects were mitigated by melatonin (Figure 6(p)). Melatonin activation of the Nrf2/Keap1 pathway represented relief of oxidative stress after PM_2.5_ treatment. Compared to the control group, Nrf2/Keap1 and SOD protein expression was significantly affected in the melatonin group but only slightly changed after PM_2.5_ exposure (Figures 6(q)–6(t)). It could therefore be concluded that the relief of oxidative stress after melatonin treatment could alleviate fat accumulation in L02 liver cells.

### 3.6. Melatonin Regulated Hepatic Lipid Metabolism through the PTP1B and Nrf2 Signalling Pathways in PM_2.5_-Treated L02 Cells

**Figure 7:**
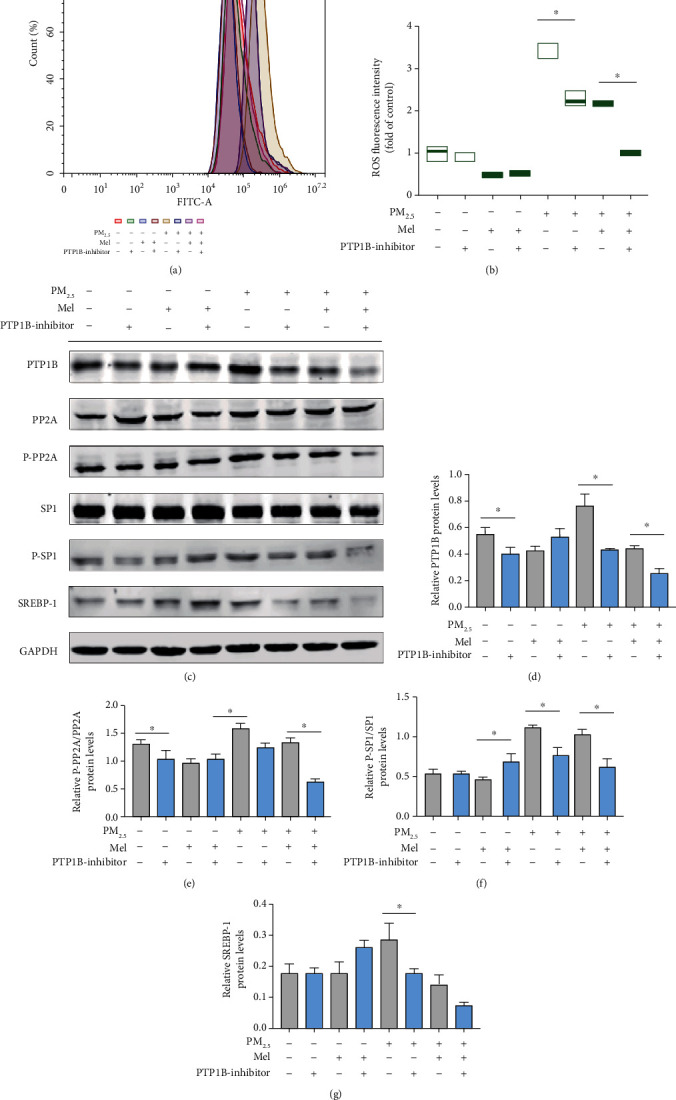
PTP1B inhibitor preconditioning eliminated lipid dysregulation in hepatocytes caused by PM_2.5_ and melatonin intervention. (a) Representative fluorescence intensity images obtained from flow cytometry in L02 cells. (b) Analysis of fluorescence intensity obtained from flow cytometry. (c) Western blotting of PTP1B, PP2A, P-PP2A, SP1, P-SP1, and SREBP-1. (d) Protein quantification of PTP1B. (e) Protein quantification of P-PP2A/PP2A. (f) Protein quantification of P-SP1/SP1. (g) Protein quantification of SREBP-1. All values are presented as the mean ± SD. ^∗^*P* < 0.05.

To confirm that PM_2.5_-induced hepatocyte steatosis occurred through the upregulation of PTP1B, a specific inhibitor of PTP1B (10 *μ*g/mL) was used in this study. Firstly, the dose was determined by PCR to detect the inhibitory effect on PTP1B mRNA expression (Supplementary Figure S3). As shown in Figures 7(a) and 7(b), both melatonin and the PTP1B inhibitor had significant ROS scavenging ability. Compared with PM_2.5_ treatment alone, melatonin treatment decreased the ROS levels by approximately 40%, and the PTP1B inhibitor decreased the ROS levels by approximately 80%. The levels of melatonin and PTP1B inhibitor in PM_2.5_-exposed cells were also significantly lower than those in the group treated with both melatonin and PM_2.5_. Similar to melatonin, the inhibitor downregulated the expression of PTP1B and its downstream molecular proteins (PTP1B, P-PP2A/PP2A, P-SP1/SP1, and SREBP-1) that regulate lipid production. The synergistic effects of melatonin and the inhibitor were greater than the effects of melatonin alone (Figures 7(c)–7(g)). Consistent with the results above, PTP1B was the direct target gene of PM_2.5_-induced oxidative stress, and inhibition of PTP1B expression downregulated the expression of its downstream gene SREBP-1 and reduced ROS production, thus reducing lipid production in L02 cells.

## 4. Discussion

Environmental PM_2.5_ has been recognized as the largest global threat affecting human health, including the development of MAFLD [4]. The pathogenesis and molecular mechanisms of PM_2.5_-induced MAFLD have not yet been well elucidated. In this study, we found that PM_2.5_ induced oxidative stress, which activated PTP1B and in turn regulated ROS release with positive feedback. Moreover, melatonin alleviated the interference of liver fat metabolism disorder caused by PM_2.5_ through regulation of the ROS-mediated PTP1B and Nrf2 signalling pathways.

MAFLD is a redefinition of NAFLD (nonalcoholic fatty liver disease). MAFLD represents a general overview of common liver metabolic disorders (not just nonalcoholics) and has multiple dominant drivers of subphenotypic response diseases. It covers more than NAFLD and has more specific diagnostic criteria [41]. Therefore, the more accurate and clear terminology of MAFLD was adopted in this work, which may provide a much wider applicability for our subsequent study to explore the toxic mechanism of PM_2.5_. In our study, ultrasonography showed that in the PM_2.5_ exposure group, the liver had a smooth contour, sharp edges, and increased parenchymal echo density, and positive contrast signs were also observed in the liver and kidney (Figure 1). The hepatorenal index is an important indicator of a fatty liver [42]. Similarly, the histopathological observation results (Oil Red O and HE staining) showed obvious fat vacuoles of varying sizes in the livers of PM_2.5_-treated mice, which presented with severe steatosis (Figure 1). Exposure to PM_2.5_ has been reported to cause systemic IR and increase the accumulation of hepatic lipids in the liver, which is consistent with our findings [43]. In addition, we observed increases in T-CHO and TAGs in mouse livers and human hepatocytes exposed to PM_2.5_, indicating that the induced lipid metabolism disorders were severe, as evidenced by the lipid index (Figures 1 and 4). The liver is a central organ for lipid homeostasis and energy metabolism [44]. Liver steatosis is caused by an imbalance in lipid homeostasis, where lipid absorption or de novo fat production exceeds lipid oxidation or output [45]. Here, our results revealed that PM_2.5_ exposure induced significant lipid accumulation in the liver accompanied by an increase in liver volume, suggesting that PM_2.5_ exposure triggered pathological changes in liver morphology and function.

To date, many studies have demonstrated that MAFLD is closely related to oxidative stress induced by PM_2.5_ exposure [5]. Oxidative stress is caused by the imbalance between the production of ROS and the ROS scavenging activity [46]. Excessive ROS results in increased adipogenesis and decreased *β*-oxidation of fatty acids, which leads to the accumulation of triacylglycerols in hepatocytes [47]. We studied the induction of ROS generation by PM_2.5_ in ApoE^−/−^ mice, and the data revealed that PM_2.5_ promoted ROS production, decreased SOD activity, and induced lipid peroxidation (as evidenced by the levels of 4-HNE and MDA) (Figure 2). Consistent with the results of the animal experiments, PM_2.5_ increased intracellular ROS in a dose-dependent manner in L02 cells (Figure 4). Compared with reliable evidence that PM_2.5_ exposure can upregulate ROS pathways, the regulation and defence of antioxidant mechanisms are relatively scarce. We further examined the mRNA and protein levels of antioxidant stress markers, namely, Nrf2/Keap1 and SOD. The qPCR results showed that PM_2.5_ acted by upregulating the Nrf2 inhibitor Keap1. Compared with the control group, Nrf2 protein expression was slightly downregulated in the PM_2.5_ group, but there was no significant difference (Figure 2). The reason for this result may be that at the transcriptional and translational levels, the course of mRNA translation into proteins is adjusted by a variety of factors, which may lead to an inconsistency between mRNA and protein expression [48]. Interestingly, the expression of Nrf2 was upregulated in the low-dose PM_2.5_ group and downregulated in the high-dose PM_2.5_ group, while the expression of Keap1 showed the opposite trend (Figure 4). It may be that the low dose of PM_2.5_ induces the body's stress response and activates Nrf2. However, with increasing exposure dose, PM_2.5_ inhibits the Nrf2 pathway. Similarly, it has been found that low concentrations of PM_2.5_ slightly upregulate Nrf2 expression, and subsequently, PM_2.5_ treatment dose-dependently decreases Nrf2 expression [10]. These studies have shown that PM_2.5_ induces ROS production and changes in antioxidant genes, which play vital regulatory roles in the progression of MAFLD.

ROS overproduction can modulate many cellular events involved in hepatic lipid metabolism diseases by regulating a variety of disease-related targets, such as PTP1B [49]. In addition, PTP1B levels were significantly elevated in the hepatocytes of fructose-fed hamsters, HFD-fed mice, and fatty liver and insulin-resistant animal models [50]. The overexpression of PTP1B in liver cells increased the expression level and transcriptional activity of SREBP1, which resulted in the increased synthesis of liver triacylglycerols and fatty acids [51]. This was due to the enhanced transcriptional activity of the recombinant SP1 site in the SREBP1 promoter by increasing the activity of PP2A when PTP1B was overexpressed [52]. This study found that PM_2.5_ could upregulate PTP1B expression by inducing ROS generation, and the expression level of PTP1B and ROS production was dose-dependent (Figures 3, 4, and 6). Especially, PM_2.5_ and melatonin did not affect the activity of PP2A and SP1 (Figure 3). This may be related to the posttranslational modification of proteins [53, 54]. NAC preconditioning inhibited PM_2.5_-induced PTP1B overexpression, suggesting that ROS plays an important role in PTP1B activation after PM_2.5_ exposure (Figure 5). Surprisingly, we found that PTP1B regulated by PM_2.5_ had positive feedback regulation on ROS release. As shown in Figure 7, pretreatment of L02 cells with PTP1B inhibitors significantly reduced ROS production and subsequently downregulated the PTP1B downstream proteins PP2A, SP1, and SREBP1. Based on previous evidence, PTP1B KO mice showed decreased ROS production and lipid peroxidation in the liver [55]. To our knowledge, our results provide new evidence of the mechanism by which PM_2.5_ exposure promotes the occurrence and development of MAFLD, demonstrating that PM_2.5_ exposure activates the ROS/PTP1B pathway and that PTP1B regulates ROS by positive feedback.

Many studies have shown that the nocturnal indole melatonin produced by the pineal gland is effective against metabolic syndrome [56]. More recently, melatonin has been shown to reverse the harmful effects of fructose in the diet, and this animal model modulates metabolic pathways such as lipid production, *β*-oxidation, lipolysis, and gluconeogenesis [57]. Melatonin may also be ingested in the liver in a dose-dependent manner through specific cellular and nuclear receptors [58]. The pathogenesis of MAFLD is complex, but melatonin may be the key to the treatment of MAFLD. Furthermore, melatonin alleviated hepatic steatosis and lipid accumulation in ApoE^−/−^ mice under different experimental conditions [59]. A previous animal study showed that ROS mediated lipopolysaccharide-induced SREBP-1c activation and lipid accumulation in the liver. Melatonin might be used as a pharmacological agent to prevent endotoxin-induced MAFLD [60]. Liver lipotoxicity is closely related to hepatic metabolic disorders caused by impaired fatty acid oxidation and increased ROS production [61]. It may be helpful to study the protective effects of melatonin on PM_2.5_-induced hepatic fatty degeneration. Therefore, more research is encouraged to explore this issue. In our study, as expected, melatonin mitigated steatosis and decreased the lipid content of the liver during PM_2.5_ damage. Both animal and cell experiments showed that melatonin effectively reduced ROS levels and helped to downregulate PTP1B and increase Nrf2 expression in PM_2.5_-treated groups, thereby changing the effects of PM_2.5_ exposure on liver lipid accumulation.

**Figure 8:**
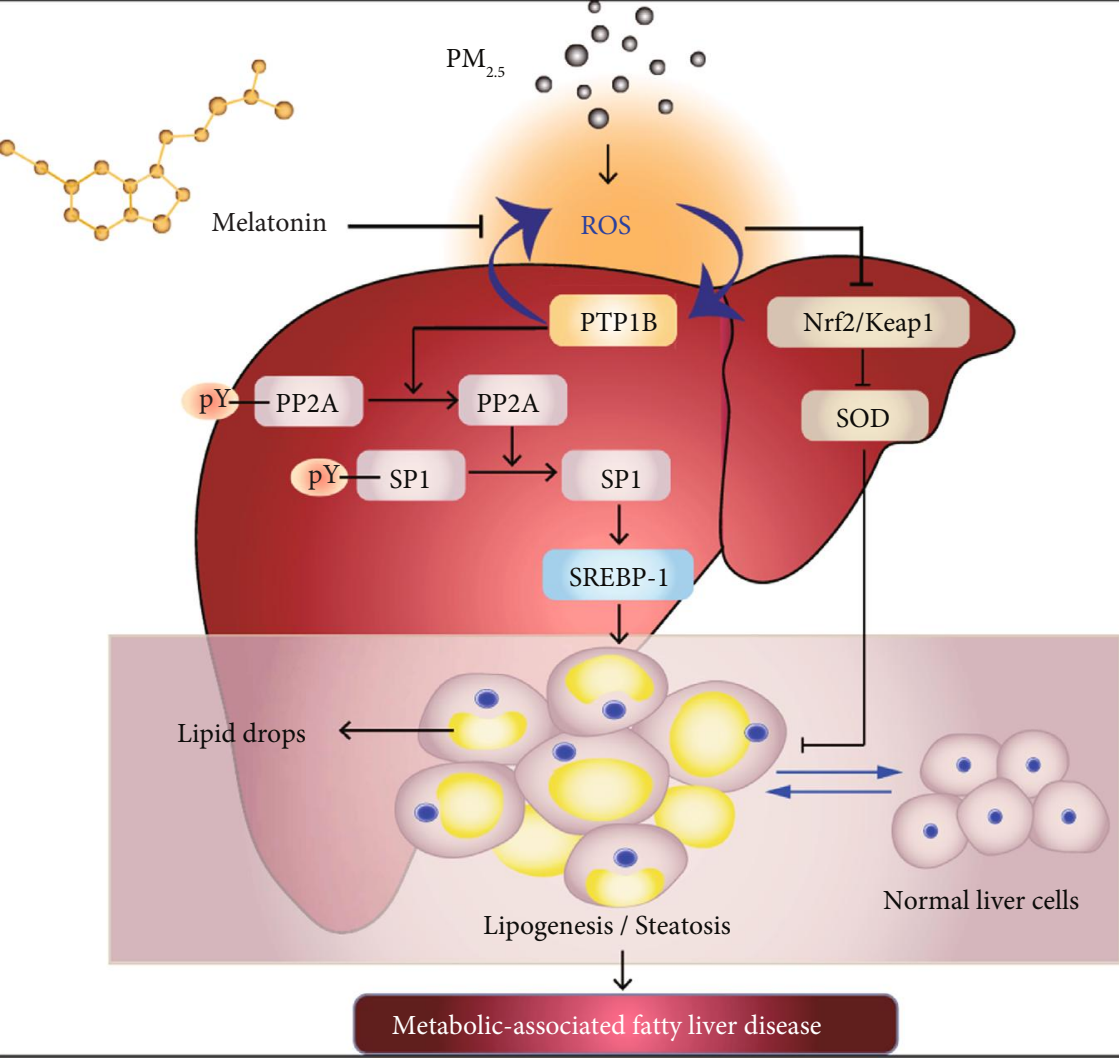
Schematic of melatonin ameliorating PM_2.5_-induced hepatic lipid accumulation.

The schematic diagram summarizing these results and mechanisms shows that PM_2.5_-induced ROS accumulation simultaneously promotes fat generation signal transduction by activating the PTP1B-PP2A-SP1-SREBP1 axis and inhibiting the Nrf2/Keap1-SOD axis, resulting in lipid accumulation and promotion of the occurrence and development of MAFLD. Moreover, melatonin plays an antioxidative stress role and regulates the ROS-mediated PTP1B and Nrf2 signalling pathways by inhibiting ROS production to alleviate the harmful effects induced by PM_2.5_ (Figure 8). A comprehensive study of ROS targets could not only provide insight into the mechanism of PM_2.5_-induced MAFLD but also give more evidence for the clinical applications of melatonin.

## 5. Conclusions

In summary, this study shows that PM_2.5_ promoted the occurrence and development of MAFLD in ApoE^−/−^ mice. Excess accumulation of PM_2.5_-induced ROS could activate PTP1B, which in turn had a positive feedback regulation effect on ROS release. Our study is the first to show that melatonin alleviated the disturbance of PM_2.5_-triggered hepatic steatosis and liver damage by regulating the ROS-mediated PTP1B and Nrf2 signalling pathways. These results suggest that melatonin administration may be a prospective therapy for the prevention and treatment of MAFLD associated with air pollution.

## Figures and Tables

**Table 1:** The primer lists of real‐time PCR.

Primer	Forward primer (5′-3′)	Reverse primer (5′-3′)
mus-SOD	AAGGGAGATGTTACAACTCAGG	GCTCAGGTTTGTCCAGAAAATG
hsa-SOD	CCCGACCTGCCCTACGACTAC	AACGCCTCCTGGTACTTCTCCTC
mus-Keap1	GACTGGGTCAAATACGACTGC	GAATATCTGCACCAGGTAGTCC
hsa-Keap1	ATTCAGCTGAGTGTTACTACCC	CAGCATAGATACAGTTGTGCAG
mus-Nrf2	CAGCCATGACTGATTTAAGCAG	CAGCTGCTTGTTTTCGGTATTA
hsa-Nrf2	TCCAAGTCCAGAAGCCAAACTGAC	GGAGAGGATGCTGCTGAAGGAATC
mus-SREBP1	GCTACCGGTCTTCTATCAATGA	CGCAAGACAGCAGATTTATTCA
hsa-SREBP1	CTGTGTGACCTGCTTCTTGT	CTCATGTAGGAACACCCTCC
mus-SP1	GAAGCAGCAGCACAGGCAGTAG	GCCAGCAGAGCCAAAGGAGATG
hsa-SP1	TCACTCCATGGATGAAATGACA	CAGAGGAGGAAGAGATGATCTG
mus-PP2A	AGTTACACTGCTTGTAGCTCTT	AACCCATAAACCTGTGTGATCT
hsa-PP2A	CGAAGGTGTGAAGGGGAAGAAGC	CAGCGTGTTGAGAAGAGCGACTAG
mus-PTP1B	GAGAGATCCTGCATTTCCACTA	TACTTTCTTGATGTCCACGGAA
hsa-PTP1B	CCATTTACCAGGATATCCGACA	TGACGTCTCTGTACCTATTTCG

## Data Availability

Most of data and materials generated or analyzed during this study are included in this manuscript. Other data are available from the corresponding authors on reasonable request.

## Supplementary Materials

Supplementary Materials Figure S1: liver damage effects of PM_2.5_. Figure S2: mRNA expression levels associated with PM_2.5_ exposure to liver injury. Figure S3: mRNA expression of PTP1B after treatment with PTP1B inhibitor. Table S1: concentrations of inorganic elements in PM_2.5_. Table S2: content of water soluble ions in PM_2.5_. Table S3: introduction of genes related to liver injury caused by PM_2.5_.
