# Supplementary material for: Melatonin Alleviates PM2.5-Induced Hepatic Steatosis and Metabolic-Associated Fatty Liver Disease in ApoE−/− Mice
Source: Oxid Med Cell Longev. 2022 Jun 8;2022:8688643. doi: 10.1155/2022/8688643 (PMC9200552; doi:10.1155/2022/8688643)
Supplement: Supplementary Materials — Figure S1: liver damage effects of PM2.5. Figure S2: mRNA expression levels associated with PM2.5 exposure to liver injury. Figure S3: mRNA expression of PTP1B after treatment with PTP1B inhibitor. Table S1: concentrations of inorganic elements in PM2.5. Table S2: content of water soluble ions in PM2.5. Table S3: introduction of genes related to liver injury caused by PM2.5. [file 8688643.f1.docx]

**Melatonin alleviates PM_2.5_-induced hepatic steatosis and metabolic-associated fatty liver disease in ApoE^-/-^ mice**

Zhou Du^1, 2^, Shuang Liang^1, 2^, Yang Li^1, 2^, Jingyi Zhang^1, 2^, Yang Yu^1, 2^, Qing Xu^3^, Junchao Duan^1, 2, *^, Zhiwei Sun^1, 2^^, *^

^1^ Department of Toxicology and Sanitary Chemistry, School of Public Health, Capital Medical University, Beijing 100069, People’s Republic of China

^2^ Beijing Key Laboratory of Environmental Toxicology, Capital Medical University, Beijing 100069, People’s Republic of China

^3^ Core Facilities for Electrophysiology, Core Facilities Center, Capital Medical University, Beijing 100069, People’s Republic of China

***Corresponding Authors**

**Zhiwei Sun,** Department of Toxicology and Sanitary Chemistry, School of Public Health, Capital Medical University, Beijing 100069, People’s Republic of China. E-mail: [zwsun@ccmu.edu.cn](mailto:zwsun@ccmu.edu.cn)

**Junchao Duan,** Department of Toxicology and Sanitary Chemistry, School of Public Health, Capital Medical University, Beijing 100069, People’s Republic of China. E-mail: jcduan@ccmu.edu.cn





**Supplementary Fig. S1. Liver damage effects of PM_2.5_.** (A) Masson staining of mice liver. (B) The proportion of fibrosis area. (C) mRNA expression levels of inflammatory cytokines in liver tissue. (D) mRNA expression levels of inflammatory cytokines in L02 cells. (E) Body weight in mice. (F) The ratio of liver weight to body weight. (G) Liver weight (g). All values are presented as the mean ± SD (n=6). **P* < 0.05 for Con group vs PM_2.5_ group and #*P* < 0.05 for PM_2.5_ group vs PM_2.5_ + Mel group.


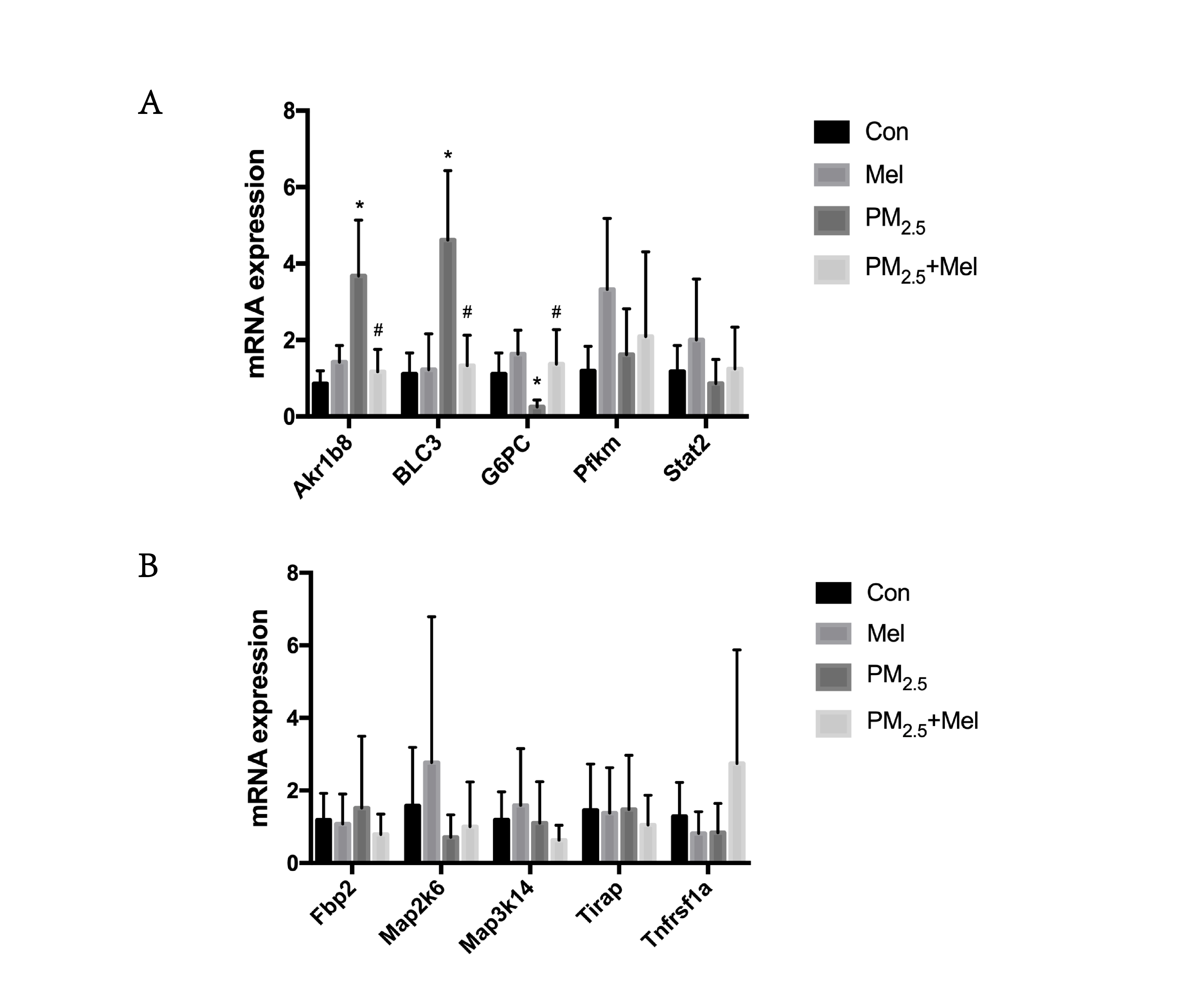


**Supplementary Fig. S2. mRNA expression levels associated with PM_2.5_ exposure to liver injury.** (A) mRNA expression in line with the expected trend. (B) mRNA expression levels not consistent with the expected trend. All values are presented as the mean ± SD (n=6). **P* < 0.05 for Con group vs PM_2.5_ group and #*P* < 0.05 for PM_2.5_ group vs PM_2.5_ + Mel group.


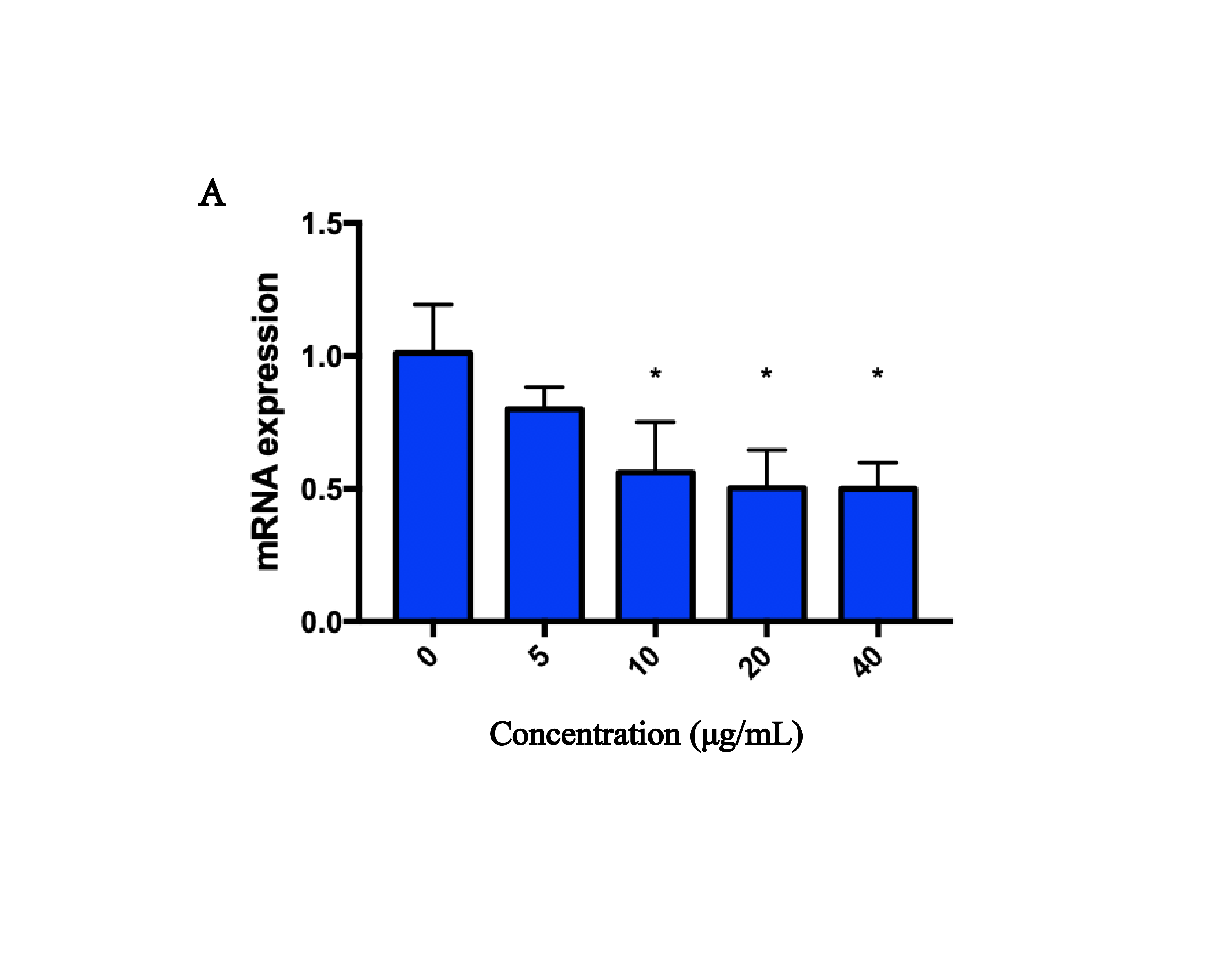


**Supplementary Fig. S3. mRNA expression of PTP1B after treatment with PTP1B inhibitor.** (A) mRNA expression of PTP1B.

**Table S1. Concentrations of inorganic elements in PM_2.5_** [1].

| Element | μg/g | Element | μg/g |
| --- | --- | --- | --- |
| S | 4000.00 | Mn | 33.50 |
| Ca | 1850.00 | Ba | 29.75 |
| Na | 1150.00 | Sn | 26.75 |
| Si | 575.00 | Cu | 13.48 |
| Fe | 455.00 | Ni | 13.43 |
| Ti | 400.00 | Sr | 10.73 |
| Mg | 300.00 | As | 5.03 |
| Zn | 146.50 | Se | 4.45 |
| K | 100.00 | Sb | 2.93 |
| Al | 92.25 | Cd | 0.68 |
| V | 75.00 | P | - |
| Cr | 40.75 | Co | - |

-, not detected.

**Table S2. Content of water soluble ions in PM_2.5_** [1].

| Anion | Concentration (mg/g) | Cation | Concentration (mg/g) |
| --- | --- | --- | --- |
| F^-^ | 0.907 | Na^+^ | 3.762 |
| Cl^-^ | 7.957 | NH_4_^+^ | 25.952 |
| NO_2_^-^ | - | K^+^ | 3.643 |
| NO_3_^-^ | 74.708 | Mg_2_^+^ | 0.812 |
| SO_4_^2-^ | 22.782 | Ca_2_^+^ | 4.626 |

-, not detected.

**Table S3. Introduction of genes related to liver injury caused by PM_2.5_**

| Gene | Expression in metabolic disorders | Function | Regulation of genes | Reference |
| --- | --- | --- | --- | --- |
| Akr1b8 | Up-regulation | Lipid synthesis, inflammation | ACC1, I1b、IFNG, TNF-α | [2] |
| Bcl3 | Up-regulation | NASH, liver lipid metabolism | NFκB1, PPARG, PPARA, PPARGC1A | [3] |
| G6pc | down-regulation | Sugar dysplasia, MAFLD | ChREBP, IRE1α, ATF4 | [4] |
| Pfkm | down-regulation | Glycolysis | FOXO3 | [5] |
| stat2 | down-regulation | Immunity, cell proliferation | NFκB1, PDLIM2 | [6] |
| Fbp2 | Up-regulation | Neoplasms, gluconeogenesis | HIF1A | [7] |
| Map2k6 | Up-regulation | Endoplasmic reticulum stress, hepatic lipid degeneration | PRKAA1, MAPK14 | [8] |
| Map3k14 | Up-regulation | Immunity, inflammation, glucagon secretion | NFκB1, CREB | [9] |
| Tirap | Up-regulation | Inflammation | CRTC | [10] |
| Tnfrsf1a | Up-regulation | Inflammation, immunity | TNF, NFκB1, IL6, TLR3, IL8 | [11] |

**References**

[1] Liu J, Liang S, Du Z, Zhang J, Sun B, Zhao T, et al. PM(2.5) aggravates the lipid accumulation, mitochondrial damage and apoptosis in macrophage foam cells. Environ Pollut. 2019;249:482-90.

[2] Shen Y, Ma J, Yan R, Ling H, Li X, Yang W, et al. Impaired self-renewal and increased colitis and dysplastic lesions in colonic mucosa of AKR1B8-deficient mice. Clinical cancer research : an official journal of the American Association for Cancer Research. 2015;21:1466-76.

[3] Gehrke N, Wörns MA, Huber Y, Hess M, Straub BK, Hövelmeyer N, et al. Hepatic B cell leukemia-3 promotes hepatic steatosis and inflammation through insulin-sensitive metabolic transcription factors. J Hepatol. 2016;65:1188-97.

[4] Rajas F, Dentin R, Cannella Miliano A, Silva M, Raffin M, Levavasseur F, et al. The absence of hepatic glucose-6 phosphatase/ChREBP couple is incompatible with survival in mice. Molecular metabolism. 2021;43:101108.

[5] Chen Y, Yu Q, Duan X, Wu W, Zeng G. Phosphofructokinase-M inhibits cell growth via modulating the FOXO3 pathway in renal cell carcinoma cells. Biochem Biophys Res Commun. 2020;530:67-74.

[6] Joyce MA, Berry-Wynne KM, Dos Santos T, Addison WR, McFarlane N, Hobman T, et al. HCV and flaviviruses hijack cellular mechanisms for nuclear STAT2 degradation: Up-regulation of PDLIM2 suppresses the innate immune response. PLoS pathogens. 2019;15:e1007949.

[7] Duda P, Janczara J, McCubrey JA, Gizak A, Rakus D. The Reverse Warburg Effect is Associated with Fbp2-Dependent Hif1α Regulation in Cancer Cells Stimulated by Fibroblasts. Cells. 2020;9.

[8] Matesanz N, Bernardo E, Acín-Pérez R, Manieri E, Pérez-Sieira S, Hernández-Cosido L, et al. MKK6 controls T3-mediated browning of white adipose tissue. Nat Commun. 2017;8:856.

[9] Sheng L, Zhou Y, Chen Z, Ren D, Cho KW, Jiang L, et al. NF-κB–inducing kinase (NIK) promotes hyperglycemia and glucose intolerance in obesity by augmenting glucagon action. Nat Med. 2012;18:943-9.

[10] Imanishi T, Unno M, Kobayashi W, Yoneda N, Akira S, Saito T. mTORC1 Signaling Controls TLR2-Mediated T-Cell Activation by Inducing TIRAP Expression. Cell reports. 2020;32:107911.

[11] Li J, Sapper TN, Mah E, Moller MV, Kim JB, Chitchumroonchokchai C, et al. Green tea extract treatment reduces NFκB activation in mice with diet-induced nonalcoholic steatohepatitis by lowering TNFR1 and TLR4 expression and ligand availability. The Journal of nutritional biochemistry. 2017;41:34-41.
